# 12/15-lipoxygenase inhibition attenuates neuroinflammation by suppressing inflammasomes

**DOI:** 10.3389/fncel.2023.1277268

**Published:** 2023-09-26

**Authors:** Canan Cakir-Aktas, Ebru Bodur, Muge Yemisci, Klaus van Leyen, Hulya Karatas

**Affiliations:** ^1^Institute of Neurological Sciences & Psychiatry, Hacettepe University, Ankara, Türkiye; ^2^Department of Medical Biochemistry, Faculty of Medicine, Hacettepe University, Ankara, Türkiye; ^3^Department of Neurology, Faculty of Medicine, Hacettepe University, Ankara, Türkiye; ^4^Neuroprotection Research Laboratory, Department of Radiology, Massachusetts General Hospital and Harvard Medical School, Charlestown, MA, United States

**Keywords:** 12/15-LOX, stroke, ischemia-recanalization, neuroinflammation, inflammasome, NLRP3, caspase

## Abstract

**Introduction:**

Lipoxygenases (LOXs) have essential roles in stroke, atherosclerosis, diabetes, and hypertension. 12/15-LOX inhibition was shown to reduce infarct size and brain edema in the acute phase of experimental stroke. However, the significance of 12/15-LOX on neuroinflammation, which has an essential role in the pathophysiology of stroke, has not been clarified yet.

**Methods:**

In this study, ischemia/recanalization (I/R) was performed by occluding the proximal middle cerebral artery (pMCAo) in mice. Either the 12/15-LOX inhibitor (ML351, 50 mg/kg) or its solvent (DMSO) was injected i.p. at recanalization after 1 h of occlusion. Mice were sacrificed at 6, 24, and 72-h after ischemia induction. Infarct volumes were calculated on Nissl-stained sections. Neurological deficit scoring was used for functional analysis. Lipid peroxidation was determined by the MDA assay, and the inflammatory cytokines IL-6, TNF-alpha, IL-1beta, IL-10, and TGF-beta were quantified by ELISA. The inflammasome proteins NLRP1 and NLRP3, 12/15-LOX, and caspase-1 were detected with immunofluorescence staining.

**Results:**

Infarct volumes, neurological deficit scores, and lipid peroxidation were significantly attenuated in ML351-treated groups at 6, 24, and 72-h. ELISA results revealed that the pro-inflammatory cytokines IL-1beta, IL-6, and TNF-alpha were significantly decreased at 6-h and/or 24-h of I/R, while the anti-inflammatory cytokines IL-10 and TNF-alpha were increased at 24-h or 72-h of ML351 treatment. NLRP1 and NLRP3 immunosignaling were enhanced at three time points after I/R, which were significantly diminished by the ML351 application. Interestingly, NLRP3 immunoreactivity was more pronounced than NLRP1. Hence, we proceeded to study the co-localization of NLRP3 immunoreactivity with 12/15-LOX and caspase-1, which indicated that NLRP3 was co-localized with 12/15-LOX and caspase-1 signaling. Additionally, NLRP3 was found in neurons at all time points but in non-neuronal cells 72 h after I/R.

**Discussion:**

These results suggest that 12/15-LOX inhibition suppresses ischemia-induced inflammation in the acute and subacute phases of stroke via suppressing inflammasome activation. Understanding the mechanisms underlying lipid peroxidation and its associated pathways, like inflammasome activation, may have broader implications for the treatment of stroke and other neurological diseases characterized by neuroinflammation.

## Graphical abstract

Effects of 12/15-LOX inhibition by ML351 on the acute and subacute phases of neuroinflammation following I/R. ROS-induced oxidative stress exacerbates the production of oxidized lipids by the 12/15-lipooxygenase enzyme in the ischemic brain. These oxidized lipids stimulate neuroinflammation and contribute to the pathophysiology of I/R. Neuroinflammation is especially driven by NLRP1 and NLRP3 inflammasome protein complexes. The inflammasomes convert procaspase-1 into its active form caspase-1. Caspase-1 facilitates the cleavage of the pro-inflammatory cytokine, IL-1beta, into its active form and leads to the release of IL-1beta from especially neurons at the acute phase of neuroinflammation. Production of other pro-inflammatory cytokines (TNF-alpha, IL-6) is significantly increased at 24 h of stroke which also initiates the synthesis of anti-inflammatory cytokines to protect the tissue itself (TGF-beta, IL-10). The 12/15-LOX inhibitor, ML351, suppresses inflammasome activation by reducing lipid peroxidation and eventually decreases infarct volume and neurological deficit score (NDS). Thus, while providing an inhibitory effect on pro-inflammatory cytokines, it also increases anti-inflammatory cytokines (A graphical abstract was created by Canan Cakir-Aktas and Hulya Karatas).

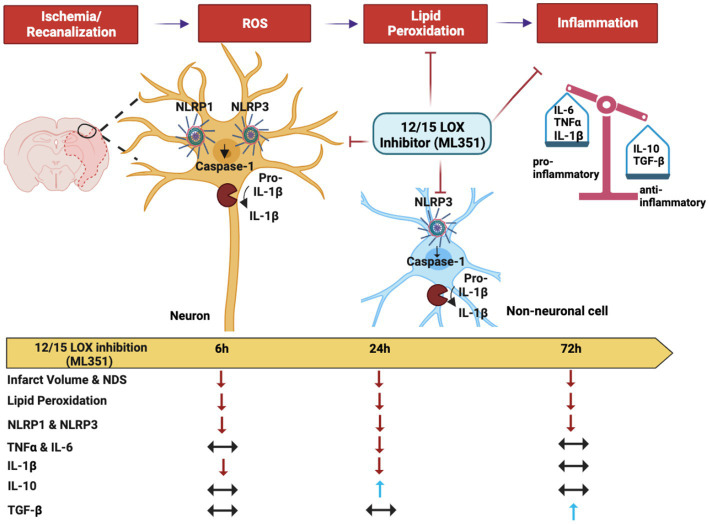

## 1. Introduction

Lipoxygenases (LOXs) are a family of lipid-oxidizing enzymes, which generate eicosanoids and related compounds from arachidonic acid and other polyunsaturated fatty acids. The 12/15-LOX is special in that it can directly oxidize lipid membranes containing polyunsaturated fatty acids, without the preceding action of a phospholipase, leading to the direct attack on organelles. This presumably underlies the cytotoxic activity of 12/15-LOX, which is upregulated in neurons and endothelial cells after stroke (van Leyen et al., 2014; Karatas and Cakir-Aktas, 2019). A major effect of 12/15-LOX on ischemic brain injury is its direct effect on lipid peroxidation, which leads to delayed cell death, blood–brain barrier (BBB) damage, and edema formation (Jin et al., 2008). Recent studies have suggested that 12/15-LOX inhibition may enhance neuroplasticity and promote neuronal survival in the post-stroke period (Yigitkanli et al., 2013). Although it has been known that 12/15-LOX inhibition has reduced infarct size and brain edema in the acute phase of the experimental stroke (Yigitkanli et al., 2013; Karatas et al., 2018), the effect of 12/15-LOX on stroke-induced neuroinflammation has not been clarified yet. Neuroinflammation is the most prominent feature of the ischemic stroke pathology (Eltzschig and Eckle, 2011; Iadecola and Anrather, 2011; Maida et al., 2020). Membrane lipid peroxidation by 12/15-LOX may induce inflammation by activating NLRP3 (Liang et al., 2021). The role of 12/15-LOX in neuroinflammation is complex and multifaceted, involving the production of pro-inflammatory mediators such as leukotrienes and reactive oxygen species. Overall, further research is needed to fully elucidate the role of 12/15-LOX in stroke pathophysiology and determine whether it represents a viable therapeutic target for stroke treatment.

Neuron cell death and especially necrotic cell debris strongly trigger inflammation following ischemic stroke (Duris et al., 2018; Puleo et al., 2022). Inflammasomes act as sensors that detect tissue damage. These complex proteins increase the production and release of pro-inflammatory cytokines such as IL-6, IL-1beta, and TNF-alpha in the peripheral tissues (Xu et al., 2021; Puleo et al., 2022). In particular, nucleotide-binding oligomerization domain (NOD)-like receptor (NLR) pyrin domain-containing 1 (NLRP1) and 3 (NLRP3) inflammasomes, are overexpressed in the brain following ischemia (Fann et al., 2018; Jiang et al., 2020; Qiu et al., 2022). After activation of NLRP3, it converts procaspase-1 into a cleaved caspase-1 (Ogura et al., 2006). Then, this activated caspase-1 triggers the inflammatory process by converting pro-IL-1beta and pro-IL 18 to their active forms. Consistently, some studies showed that inhibition of NLRP-3 inflammasome has reduced the ischemia/recanalization (I/R) injury and protected the BBB in both *in vivo* and *in vitro* ischemic conditions (Abulafia et al., 2009; Gao et al., 2017; Bellut et al., 2021). Therefore, this study aimed to investigate the role of 12/15-LOX inhibition by a novel and potent 12/15-LOX inhibitor, ML351, on the inflammasome-related neuroinflammation induced by ischemia in acute and subacute phases of the MCAo stroke model in mice.

## 2. Materials and methods

### 2.1. Animal procedure and experimental groups

A total of 77 male Swiss Albino mice aged 8–12 weeks, weighing 30–40 grams, were used in the experiments. All experimental procedures were approved by Hacettepe University, Animal Experiments Ethics Committee (Approval No: 2016/27–04). Researchers performing the experiments were blinded to the experimental groups. The mice were randomly divided into three groups: (a) The naive group, which did not undergo ischemia; (b) the 12/15-LOX inhibitor (ML351)-treated group; and (c) the vehicle (DMSO)-treated group. Mice were anesthetized with 4% isoflurane inhalation before surgery and maintained at 1–2% isoflurane during the procedure. The body temperature of the mice was kept at 37 ± 0.2° C with the rectal probe of the homeothermic blanket (Physiosuit, Kent Scientific, United States). Oxygen saturation and heart rate were monitored during the surgery with a pulse oximeter (V3304 Digital Table-Top Pulse Oximeter, Nonin, United States). Ischemia/recanalization was induced by proximal Middle Cerebral Artery Occlusion (pMCAo) using the intraluminal filament method. Briefly, the common and external carotid arteries were exposed through an incision employed in the middle of the neck and permanently ligated with sutures. The main carotid artery was pushed through a filament leading into the internal carotid artery with an incision made in its middle part, and the rounded end of the filament was pushed until it reached the middle cerebral artery (Park et al., 2014). Regional cerebral blood flow (rCBF) was monitored with a Laser Doppler flowmeter (AD Instruments, New Zealand) by a flexible probe placed on the skull (2 mm posterior, 6 mm lateral to the bregma) to determine whether ischemia was induced and whether successful occlusion was counted if rCBF decreased below 30% compared to the basal condition. Mice were re-anesthetized 1-h after the occlusion, and the filament was removed. Either the newly synthesized 12/15-LOX inhibitor ML351 (50 mg/kg) or its solvent DMSO was injected intraperitoneally at recanalization after 1-h of occlusion (Rai et al., 2014). Mice were sacrificed under high-dose chloral hydrate anesthesia at 6, 24, or 72 h after recanalization.

### 2.2. Assessment of functional neurological deficit score

The following scoring system was employed for baseline and postoperative neurological examinations (Bederson et al., 1986). According to this grading, 0: no visible neurological damage (normal); 1: inability to extend the right paw (mild); 2: turn to the opposite side (middle); 3: loss of walking or righting reflex (severe).

### 2.3. Infarct volume measurement

Nissl-staining was used to assess the ischemic brain damage. Mice were perfused transcardially with heparin and 4% paraformaldehyde (PFA) solution sequentially. Then the brains were removed and fixed in 4% PFA overnight at 4°C. Then, they were sectioned into 2-mm-thick coronal slices and embedded in paraffin (*n* = 3 mice/group). Each of the blocks was cut into 5 μm thick sections. After deparaffinization, the slides were first placed in 100, 95, and 80% ethanol solutions for 30 s each. Following this, the sections were incubated with 0.1% cresyl violet solution for 3 min at 37°C. Immediately after incubation, they were placed into 95% ethyl alcohol solution for 2 min and the excess staining was removed. Afterward, the sections were covered by an entellan mounting medium following incubation with xylene for 2 min. Images were taken with a light microscope at 1X magnification. The infarct volume was calculated by multiplying five sequential infarcted areas by the section thickness of 2 mm on the posterior surface of each coronal slice (Renolleau et al., 1998; Rousselet et al., 2012; McBride et al., 2015).

### 2.4. Analysis of lipid peroxidation (MDA)

Lipid peroxidation was measured by TBARS Assay Kit (OxiSelect TBARS Assay Kit, CellBiolabs). The principle of this method is based on the formation of a 1:2 conjugate of Malondialdehyde (MDA) with thiobarbituric acid (TBA). First, the tissue samples were diluted with 5% butyl hydroxytoluene, and 100 μL of sodium dodecyl sulfate (SDS) lysis solution was added to 100 μL of the sample and incubated at room temperature for 5 min. After that, 250 μL TBA solution was added to samples, and the mixtures were incubated at 95°C for 45 min. Thereafter, the mixtures were cooled on ice for 5 min and centrifuged at 10.000 x g for 15 min. Then, 200 μL of the supernatant was taken and transferred to a new tube, and 300 μL butanol was added. The mixture was vortexed at 3000 rpm for 3 min and centrifuged at 10.000 x g for 5 min. The absorbances of the supernatants were read at a wavelength of 532 nm in a microplate reader (SpectraMax M2, Molecular Devices, United States). A standard graph was employed to calculate the results. The results were standardized by dividing the wet tissue weight.

### 2.5. Tissue preparation for the biochemical analysis

Following model formation, the mice were sacrificed with high-dose anesthesia, and the fresh brain tissues were removed. After the removal of the infarcted brain region, the tissues were weighed. A buffer (25 mM Tris buffer (pH 7.4; 2 mM EDTA, 5 mM MgCl_2_, 0.1% Triton X-100)) suitable for the biochemical analyses was used for tissue homogenization. After adding buffer and protease inhibitor (1X) to the tissues, they were homogenized at 10% (w/v) by Ultra-Turrax^®^ (S8N-5 g, IKA-Werke GmbH) on ice for 3 × 10 s. All these procedures were performed on ice to prevent protein degradation. For MDA analysis, 100 μL samples were aliquoted from each homogenate and stored in a − 80°C freezer. The remaining homogenates were centrifuged at 13.000 g for 15 min at +4°C. After centrifugation, the supernatant was separated from the pellet and used for ELISA.

### 2.6. Quantitative analysis of cytokines with enzyme-linked immunosorbent assay (ELISA)

Quantitative expression of inflammatory cytokines (IL-6, TNF-alpha, IL-1beta, IL-10, and TGF-beta) in the ischemic brain regions was detected by the sandwich ELISA method. IL-6 (Biolegend, United States), TNF-alpha (Biolegend, United States), IL-1beta (Thermo Fisher, Germany), IL-10 (Biolegend, United States), and TGF-beta (Biolegend, United States) were quantified with ELISA kits according to the manufacturer’s protocols. The levels of cytokines in naive, I/R-DMSO and I/R-ML351-treated brain samples were compared. Three mice in each group were analyzed for ELISA, and each sample was tested in duplicate. The absorbances of the samples were read at a wavelength of 532 nm on a microplate reader (SpectraMax M2, Molecular Devices, United States). The curve of the standard graph was employed to calculate the results. The results were normalized by the amount of total protein, which was determined by BCA assay in the samples (ng/mg protein).

### 2.7. Immunofluorescence method

For the immunohistochemical staining, mice were transcardially perfused with heparinized saline along with 4% PFA at 6, 24, and 72-h after ischemia (*n* = 6 mice/group). The brain tissues were carefully removed and fixed in PFA at 4°C overnight. Subsequently, the brains were sectioned in the coronal plane at a thickness of 12 μm, following overnight incubation in a 30% sucrose solution. The tissue sections were incubated in sodium citrate buffer (pH 6) at 80°C for 15 min for antigen retrieval. For the blocking step, they were incubated with normal serum of the associated secondary antibody host (10%) and BSA 1% in TBS for 1 h RT. Next, the sections were incubated with primary antibodies targeting specific proteins, namely NLRP1 [NALP1 Antibody (M-90), Santacruz; 1:100], NLRP3 [anti-NLRP3/NALP3, mAb (Cryo-2), Adipogen; 1:100], 12/15-LOX (kindly provided by Klaus van Leyen) and caspase-1 (Abcam; 1:200), at 4°C overnight. Following the primary antibody incubation, the sections were washed three times with TBS containing 0.025% Triton X-100 (Merck Millipore, 1,086,031,000). Subsequently, the sections were incubated with appropriate Cy-3 or Alexa Fluor 488 conjugated anti-mouse or anti-rabbit IgG secondary antibodies (Jackson ImmunoResearch 115–165-146 or 115–545-146; 111–165-144 or 111–545-144). To employ double immunostaining of NLRP1/NeuN, NLRP3/NeuN, NLRP1/Iba1, NLRP3/S100 beta, 12/15-LOX/ NLRP3, and caspase-1/NLRP3, the blocking step was repeated using a 10% normal serum of the associated secondary antibody host, BSA 1% in TBS for 1-h at RT. Thereafter, the sections were incubated with primary antibodies targeting specific proteins, including NeuN (Millipore; 1:200), Iba-1 (Novus; 1:200), and ALDH1 (Abcam, 1:200) at 4°C overnight. Following the washing step, the sections were incubated with appropriate Cy-3 or Alexa Fluor 488 conjugated secondary antibodies (Jackson ImmunoResearch). Finally, the stained tissues were carefully mounted with a PBS/glycerol medium containing Hoechst 33258 (InvitrogenTM) to visualize the cellular nuclei. For each tissue section, three images were captured from the peri-infarct area using a Leica TCS SP8 confocal laser scanning microscope (Leica, Wetzlar, Germany). To quantify the immunofluorescence labeling results, the number of NLRP1 and NLRP3 positive cells was counted by researchers who were blinded to the DMSO and LOX inhibitor treatment groups in the I/R model. The captured images were then analyzed using Image J software (NIH, Bethesda, MD, United States). To ensure accuracy and eliminate bias, the results were standardized by calculating the proportion relative to the counts obtained from naive brain tissue.

### 2.8. Statistics

Data were presented as mean ± standard error of the mean (S.E.M.). Differences among experimental groups were analyzed by student’s *t*-test or ANOVA followed by Tukey’s *post hoc* test. Non-normally distributed data of groups were compared using the Kruskal-Wallis test, and the Mann–Whitney U test was used for two-group comparisons as a *post hoc* test. A *p*-value ≤0.05 was considered statistically significant. Statistical analyses were carried out using GraphPad Prism software version 6.

## 3. Results

### 3.1. 12/15-LOX inhibition decreases tissue damage by suppressing lipid peroxidation, infarct size, and production of pro-inflammatory cytokines

**Figure 1:**
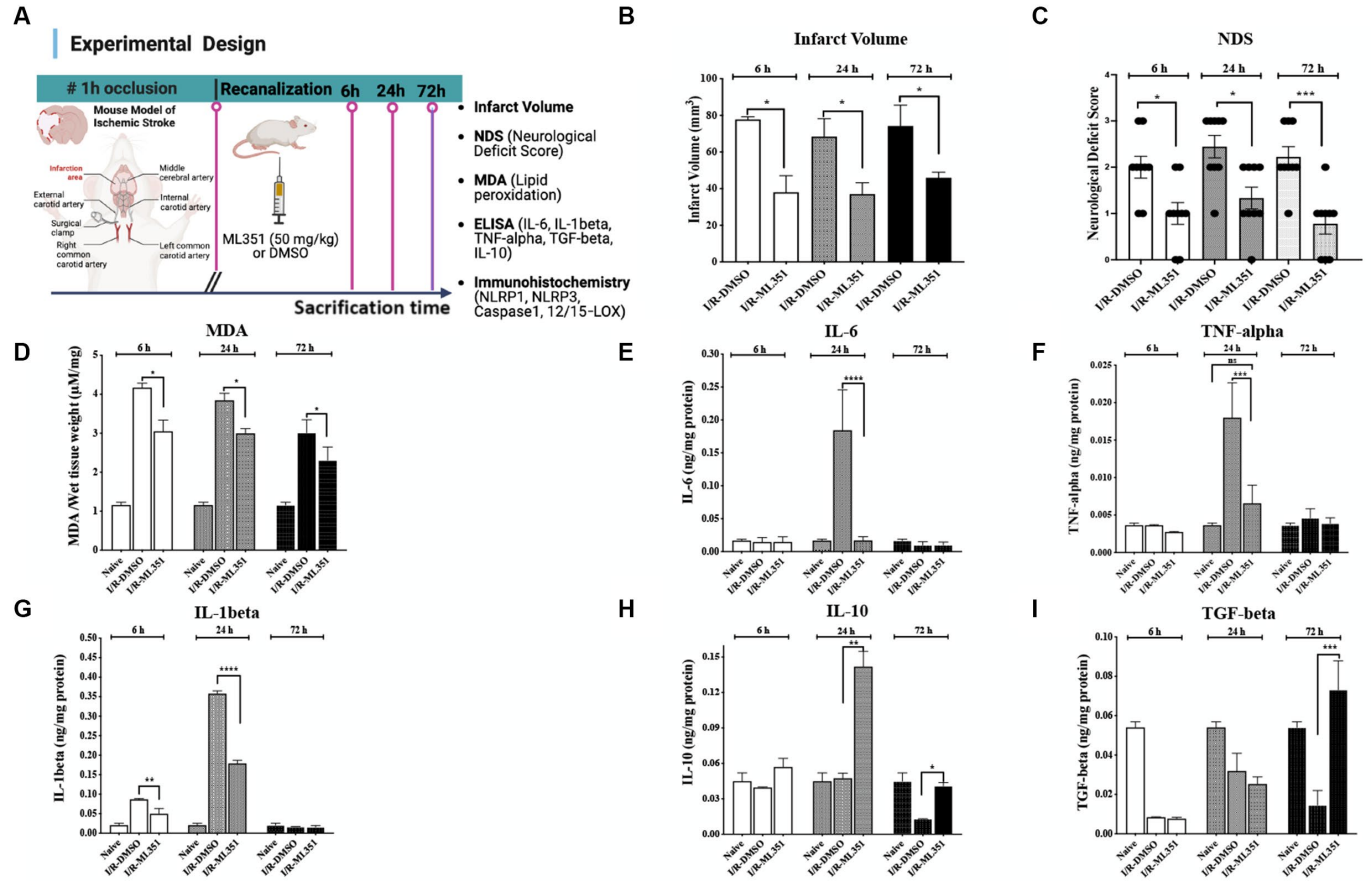
Determination of infarct volume, neurological deficit, lipid peroxidation, and pro- and anti-inflammatory cytokine levels in the brain tissues of I/R-DMSO and I/R-ML351 mice. **(A)** The experimental design of the study is summarized in the schema. Briefly, ischemia/recanalization (I/R) was performed by proximal middle cerebral artery occlusion in mice. Either the 12/15-LOX inhibitor (ML351, 50 mg/kg) or its solvent, DMSO, was injected i.p. at recanalization after 1-h of occlusion. Mice were sacrificed at 6, 24, and 72-h after ischemia induction. **(B)** Infarct volume analysis was performed on Nissl-stained sections. Infarct volumes were significantly decreased in ML351-treated groups at 6, 24, and 72-h (*n* = 3 mice/group, ^*^p ≤ 0.05, ^*^p = 0.021, ^*^p ≤ 0.050, respectively). **(C)** Functional outcome was determined with neurological deficit scoring following I/R. Neurological deficit score results showed that 12/15-LOX inhibition significantly improved neurological deficit at all time points (*n* = 9 mice/group, ^*^p = 0.0420 for 6-h; ^*^p = 0.0170 for 24-h; ^***^p = 0.0008 for 72-h). **(D)** MDA analysis results showed that increased lipid peroxidation after I/R was attenuated with ML351-treatment at all time points (*n* = 3 mice/group, ^***^p = 0.0004 for 6-h, ^*^p = 0.0196 for 24-h, ^*^p = 0.0414 for 72-h). **(E–G)** Quantitative analysis of IL-6, TNF-alpha, and IL-1beta pro-inflammatory cytokines was done with ELISA (*n* = 3 mice/group), data is given as proportioned to the total protein levels of the brain tissue samples (ng/mg protein). Pro-inflammatory cytokines were induced especially at 24-h following I/R. IL-6 (^****^p < 0.0001) and TNF-alpha (^***^p < 0.0004) were suppressed by administration of ML351 at 24-h of ischemia. IL-1beta was increased at 6-h (^***^p = 0.0002) and 24-h (^****^p < 0.0001) of I/R and decreased with ML351 treatment at 6-h (^**^p = 0.003) and 24-h (^****^p < 0.0001). **(H,I)** Quantitative analysis of anti-inflammatory cytokines, IL-10, and TGF-beta, with ELISA (*n* = 3 mice/group). ML351 induced the IL-10 anti-inflammatory cytokine at 24-h and 72-h of I/R (^**^p = 0.0069, ^*^p = 0.0192, respectively), while TGF-beta was increased at 72-h of I/R by ML351 treatment (^***^p = 0.0002).

The study investigated the effects of 12/15-LOX inhibition with ML351 on tissue damage and inflammation in an ischemia/reperfusion (I/R) mouse model. The experimental design of the study is summarized in Figure 1A. To examine the tissue damage induced by ischemia/recanalization, infarct volumes were determined with Nissl staining. 12/15-LOX inhibition significantly reduced infarct volume at 6, 24, and 72 h of I/R (*p* ≤ 0.05; from 77.8 ± to 38.0 ± mm^3^; *p* = 0.021, from 68.4 ± to 37.0 ± mm^3^; *p* ≤ 0.050, from 74.2 ± to 46.0 ± mm^3^, *n* = 3 mice/group, respectively) (Figure 1B). Neurological deficit score results were in parallel with infarct volume reduction and showed that 12/15-LOX inhibition significantly improved neurological deficit at all time points (*p* = 0.042 for 6-h; *p* = 0.017 for 24-h; *p* = 0.0008 for 72-h) (Figure 1C). Next, an MDA assay to determine lipid peroxidation following the acute and subacute phases of I/R was performed. MDA assay results showed that increased lipid peroxidation activity was significantly attenuated in ML351-treated groups at all time points (*p* = 0.0004, 6-h; *p* = 0.0196, 24-h; *p* = 0.0414, 72-h) (Figure 1D). Infarct volume, neurological deficit score, and MDA analysis data were in line with the previous findings (Yigitkanli et al., 2013; Karatas et al., 2018).

The oxidative degradation of membrane lipids following ischemic conditions leads to the production of pro-inflammatory cytokines and results in an imbalance between anti- and pro-inflammatory cytokines (Perini et al., 2001). Based on this information, we studied pro-inflammatory (IL-6, TNF-alpha, and IL-1beta) and anti-inflammatory (IL-10 and TGF-beta) cytokines with ELISA to clarify whether there were any corresponding changes between them during different stages of ischemia. ELISA results obtained from infarct and peri-infarct areas revealed that pro-inflammatory cytokines IL-6 and TNF-alpha parallelly and significantly increased at 6 or 24-h of I/R, which were suppressed by ML351 treatment (*p* < 0.0001; p = 0.0004) (Figures 1E,F). IL-1beta is the most prominent pro-inflammatory cytokine in the inflammasome pathway, which is activated by inflammasome protein complexes. It is significantly increased at 6-h (*p* = 0.0002) and 24-h (p < 0.0001) of I/R and decreased by ML351 treatment in this study (*p* = 0.03 at 6-h and *p* < 0.001 at 24-h) (Figure 1G).

The homeostatic status of tissue is characterized by a balance between pro- and anti-inflammatory cytokines. It is known that this balance is disrupted after a stroke (Perini et al., 2001). Hence, we next analyzed the anti-inflammatory cytokines, IL-10, and TGF-beta with ELISA. IL-10 results displayed that IL-10 increased at 24-h and 72-h in ML351-treated groups which were more pronounced at 24-h (*p* = 0.0069 for 24-h, *p* = 0.0192 for 72-h) (Figure 1H). One of the most prominent anti-inflammatory cytokines, TGF-beta was remarkably decreased compared to naive brains in DMSO-administered I/R groups at all time points; however, ML-351 treatment significantly increased TGF-beta levels only at 72 h of I/R (p = 0.0002) (Figure 1I).

These findings suggest that ML351 treatment plays a crucial role in maintaining the balance between pro- and anti-inflammatory cytokines, which may contribute to its protective effects in reducing tissue damage and neuroinflammation. The prominent increases in IL-10 and TGF-beta levels at 24-h or 72-h respectively, and the significant increase in TGF-beta levels at 72-h provide suggestive evidence that ML351 may have a delayed but potent effect on promoting anti-inflammatory responses. To further explore I/R-induced neuroinflammation, we investigated the involvement of inflammasomes in ischemia-induced tissue damage.

### 3.2. NLRP1 inflammasome is decreased via ML351 administration in the acute and subacute phases of I/R

**Figure 2:**
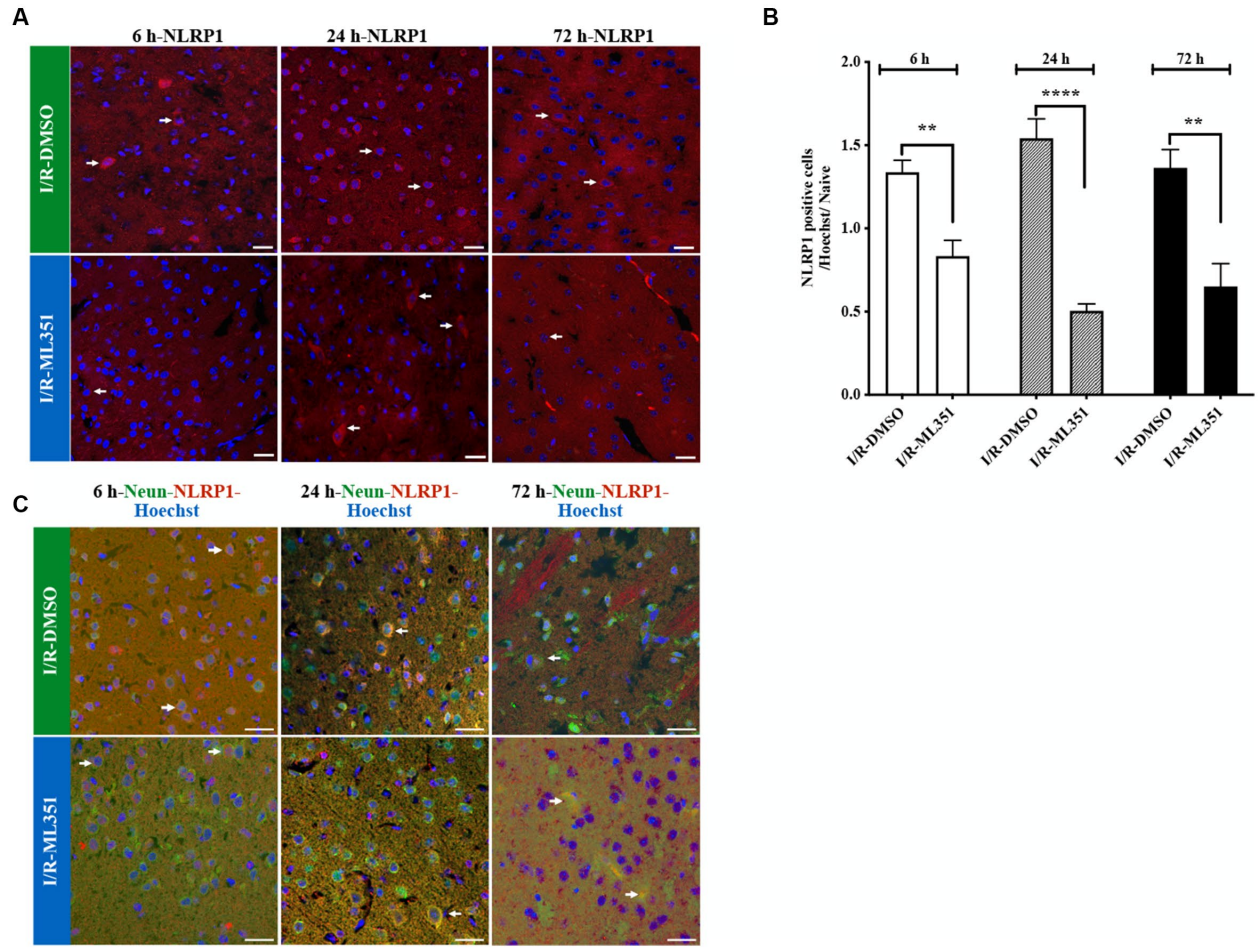
Immunofluorescence analysis of NLRP1 inflammasome at 6-h, 24-h, or 72-h following I/R. **(A)** NLRP1 inflammasome protein was detected with immunofluorescence staining (*n* = 5 mice/group). Images of Hoechst-33258-labeled cell nuclei (blue) were overlapped with the images of NLRP1 (40X, scale bar = 25 μm). NLRP1 inflammasome complex, whose expression was increased in the cell cytoplasm 6, 24, or 72-h after I/R, was decreased by 12/15-LOX inhibition (ML351). In the ML351 treated group at 24-h after I/R, NLRP1 appears to be expressed in both the soma and its axonal extension, which may indicate that it is neuronal (White arrow). **(B)** Quantitative analysis showed that NLRP1 increased in DMSO groups at 6-h (^**^p = 0.002), 24-h (^****^p < 0.0001), and 72-h (^**^p = 0.0013) (n = 3 mice/group) and decreased with ML351 treatment. **(C)** Double labeling of NLRP1 (red) and neurons (NeuN; neuronal marker) (green) showed that NLRP1 is colocalized with the NeuN signal. Some of the overlapping markings are shown with arrows.

Inflammasomes act as sensors that detect tissue damage, including cerebral ischemia. In this study, we examined the effects of 12/15-LOX inhibition with ML351 on NLRP1 and NLRP3 at all time points following I/R. The results showed that NLRP1 inflammasome labeling was increased in the acute and subacute phases (6, 24, and 72 h) after I/R in the DMSO-treated groups compared to naive brains. However, ML351 treatment significantly decreased NLRP1 inflammasome activation at all time points (at 6-h, *p* = 0.002; at 24-h, *p* < 0.0001, and at 72-h, *p* = 0.0013). The staining character of NLRP1 was mostly cytoplasmic (Figures 2A,B, white arrow). We performed double staining to show the NLRP1-positive cell types and demonstrated that NLRP1 was mostly colocalized with the neuronal marker (NeuN) (Figure 2C). This suggested that neuronal NLRP1 inflammasome activation contributes to the inflammatory response following cerebral ischemia. Additionally, the decrease in NLRP1 immunoreactivity with ML351 treatment indicated that 12/15-LOX inhibition may have a protective effect against inflammasome-mediated inflammation in this model.

### 3.3. ML351 treatment attenuates NLRP3 positive cells after acute and subacute phases of I/R

**Figure 3:**
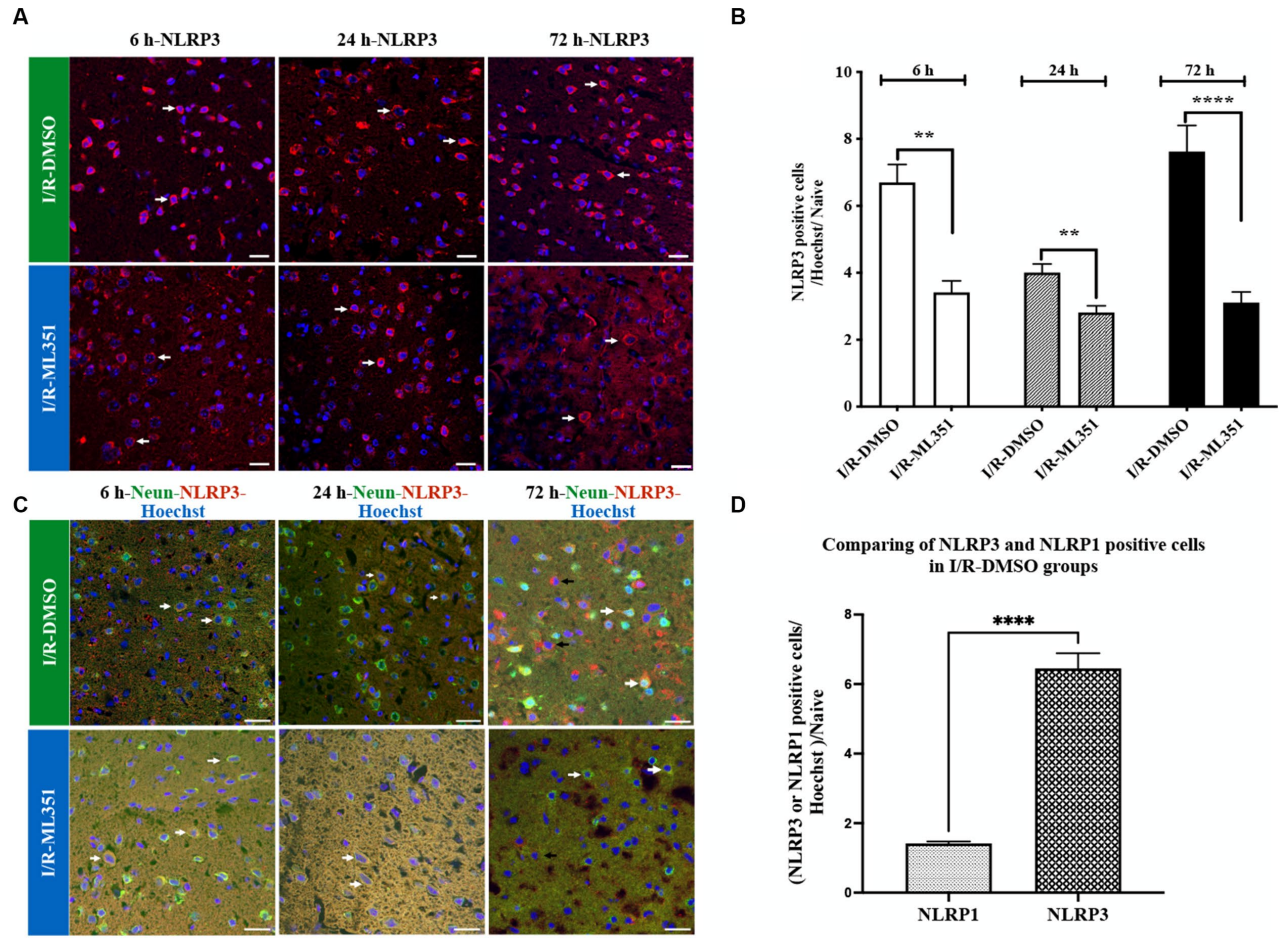
Immunofluorescence analysis of NLRP3 inflammasome at 6-h, 24-h, or 72-h following I/R. **(A)** Immunofluorescence staining of NLRP3. Nuclei were stained with Hoechst. Representative images of NLRP3 (red) and Hoechst-33258 (blue) were overlapped (40X, scale bar = 25 μm). Cytoplasmic NLRP3 labeling was increased in the DMSO groups of the acute and subacute phases of I/R. By the administration of ML351, labeling and signal intensity of NLRP3 were dramatically decreased at all time points following I/R. **(B)** Graphical representation of the changes in cell numbers with positive NLRP3 immunoreactivity at 6, 24, and 72-h. NLRP3 positive cell numbers were increased in both the acute (6 and 24-h) and subacute (72-h) phases of I/R. The decreases in NLRP3 immunoreactivity at 6-h (^***^p = 0.0003), 24-h (^**^p = 0.0027) and 72-h (^****^p < 0.0001) with ML351 treatment were statistically significant (*n* = 3 mice/group). **(C)** Double immunolabeling of NLRP3 (red) and neurons (NeuN; neuronal marker) (green) showed that NLRP3 is colocalized with NeuN (white arrows) signal especially at 6-h and 24-h after I/R. At 72-h of I/R NLRP3 immunoreactivity was present both in neurons (white arrows) and non-neuronal cells (black arrows). **(D)** Comparison of NLRP3 and NLRP1 staining in I/R-DMSO groups normalized to naive brain tissue. NLRP3 immunostaining was significantly increased compared to NLRP1 immunoreactivity (^****^p < 0.0001).

NLRP3 immune signaling increased with I/R at three-time points, prominently 6-h and 72-h, and was significantly diminished by ML351 administration (at 6-h, *p* = 0.0003; 24-h, *p* = 0.0027; 72-h, *p* < 0.0001) (Figures 3A,B). These results demonstrate that NLRP3 plays a crucial role in the inflammatory response following I/R, and the complex activation kinetics of NLRP3 should be investigated in further studies. NLRP3 inhibition by ML351 could potentially be a therapeutic strategy for reducing inflammation. Furthermore, the significant decrease in NLRP3 signaling at 6-h and 24-h with ML351 highlights the importance of early intervention in mitigating neuronal inflammasome activation. To examine the cellular source of NLRP3, double staining was performed with NLRP3 and the neuronal cell marker, NeuN, which showed that NeuN-labeled neurons were immunopositive for NLRP3 (Figure 3C). Interestingly, NLRP3 presence was determined in non-neuronal cells at 72-h of I/R (Figure 3C). This change in cellular source suggests that NLRP3 may play a role in both neuronal and non-neuronal cells during the progression of I/R. Further investigation is needed to determine the specific functions of NLRP3 in these different cell types and its implications for stroke pathology.

**Figure 4:**
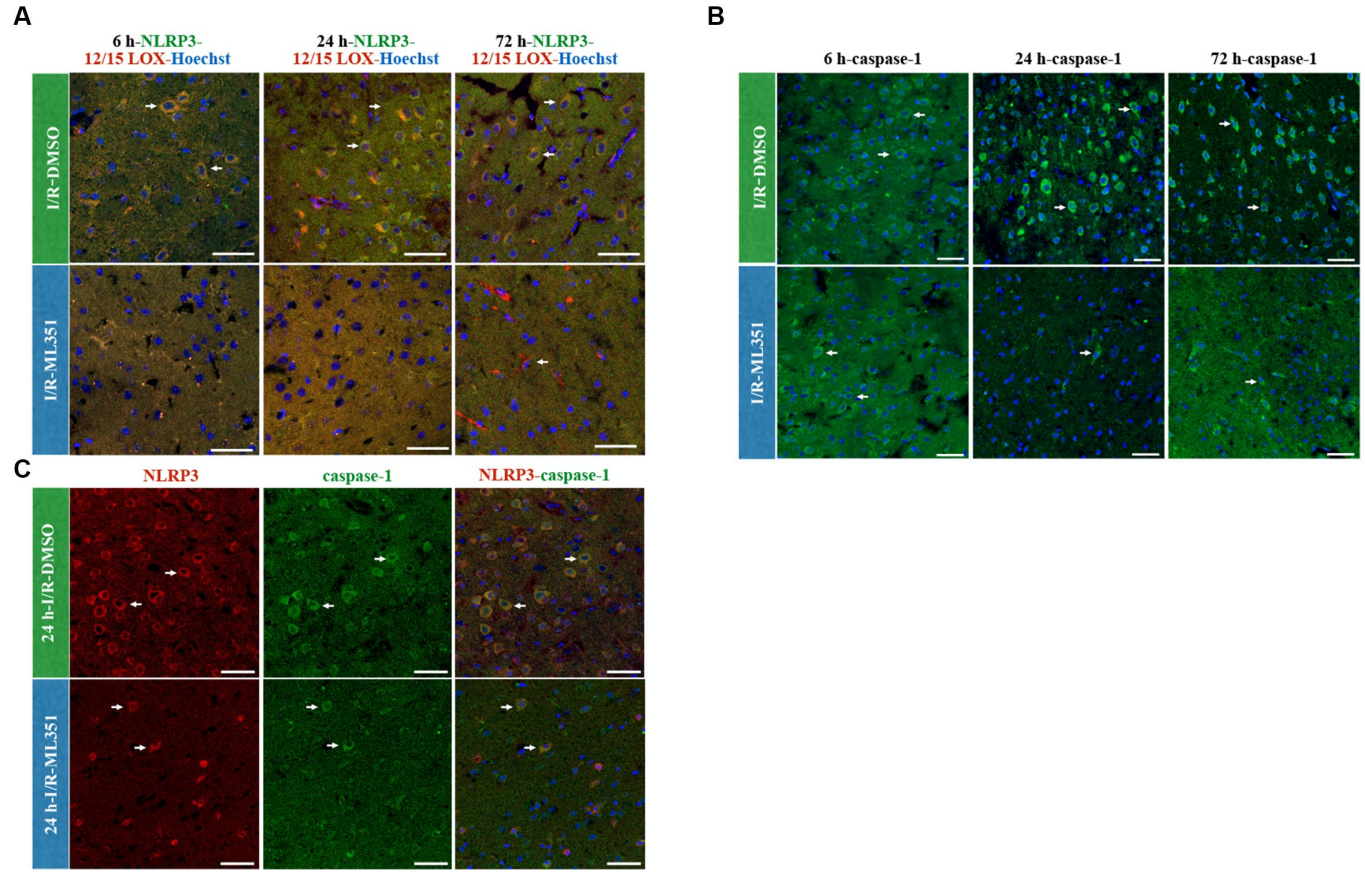
NLRP3/12/15-LOX, Caspase-1, and NLRP3/Caspase-1 immunolabelling at 6-h, 24-h, or 72-h following I/R. Nuclei were stained with Hoechst-33258 (blue) (40X, scale bar = 25 μm). **(A)** Representative images of NLRP3 (green), 12/15-LOX (red), and Hoechst-33258 (blue) were overlapped in the third panel (*n* = 3). Double labeling of NLRP3 and 12/15-LOX showed that there was a strong colocalization that acted in parallel and decreased in ML351-treated brains at all time points of I/R. **(B)** Caspase-1, a downstream effector of inflammasome activation, staining indicated that caspase-1 is increased mainly at 24-h and 72-h of I/R. **(C)** NLRP3 and caspase-1 double labeling was performed at 24-h of I/R brains, as prominent caspase-1 activation was observed at this time point, which revealed that caspase-1 positive cells were also positive for NLRP3.

Interestingly, the study demonstrated that NLRP3 activation was more pronounced than NLRP1 activation according to the percentage of cell counting results of DMSO-treated groups (Figures 2B, 3B). The ratio of NLRP3 positive cells to all cells (Hoechst positive) was approximately 6.44 times higher in DMSO-treated groups compared to naive brain tissue, while the ratio of NLRP1 positive cells to all cells was only approximately 1.42 times higher (*p* < 0.0001) (Figure 3D). This significant increase in NLRP3-positive cells indicates that NLRP3 inflammasome activation is more robust and widespread in response to ischemic injury. Hence, we proceeded to study the co-localization of the NLRP3 inflammasome with 12/15-LOX staining. NLRP3 was positive in cells expressing 12/15-LOX both in the acute and subacute phases of ischemia-recanalization (Figure 4A). Through the administration of the 12/15-LOX inhibitor ML351, the expression of NLRP3 and 12/15-LOX dramatically decreased (Figure 4A). Our data showed that ML351, an inhibitor of 12/15-LOX, suppressed neuroinflammation by inhibiting the NLRP3 inflammasome at 6-h, 24-h, and 72-h following I/R.

When an inflammasome complex assembles, caspase-1 is activated via the cleavage of procaspase-1. We showed that caspase-1 was increased after I/R (Figure 4B), which was compatible with ELISA results of proinflammatory cytokines, IL-1beta, IL-6, and TNF-alpha. The caspase-1 increase was mostly prominent at 24-h and 72-h of I/R groups, which were dramatically suppressed with the administration of ML351. Formerly, we performed double immunostaining of caspase-1 and NLRP3 inflammasome. All the NLRP3-positive cells expressed caspase-1 with 24-h of I/R was the greatest signal increase observed (Figure 4C). As expected, NLRP3 and caspase-1 expression were suppressed with the administration of ML351 (Figure 4C). These results suggested that the increased expression of caspase-1 in the I/R groups was closely associated with the activation of the NLRP3 inflammasome, which was effectively inhibited by the administration of the 12/15-LOX inhibitor. Altogether, these findings highlight the potential therapeutic benefits of targeting the 12/15-LOX pathway to reduce neuroinflammation via the inflammasome pathway and improve outcomes in ischemic stroke which were summarized in the graphical abstract figure.

In conclusion, the findings of this study suggest that ML351, a 12/15-LOX inhibitor, exerts protective effects in an I/R mouse model by reducing tissue damage, suppressing lipid peroxidation, and modulating the production of pro- and anti-inflammatory cytokines. These effects are, at least in part, mediated by the inhibition of NLRP1 and NLRP3 inflammasome activation, which may play a crucial role in the inflammatory response following cerebral ischemia. Targeting the 12/15-LOX pathway and inflammasome activation could be a potential therapeutic strategy for reducing neuroinflammation and improving outcomes in ischemic stroke.

## 4. Discussion

Cerebral I/R causes tissue damage via triggering lipid peroxidation as well as neuroinflammation (Haeggstrom et al., 2010). Several studies indicate that there is an increase in the amount of free arachidonic acid and the lipoxygenase enzyme which uses arachidonic acid as a substrate, also increases in ischemic brain tissue (Tong et al., 2002). Accordingly, the widespread expression of 12/15-LOX protein in both neurons and endothelial cells surrounding the infarct area has been shown in the literature (Jin et al., 2008). Furthermore, it has been shown that 12/15-LOX enzyme activity increased in the ischemic mouse brain (Jin et al., 2008). A major effect of the 12/15-LOX enzyme on ischemic brain injury is its direct effect on lipid peroxidation, which leads to delayed cell death, blood–brain barrier damage (BBB), and edema formation in the penumbra (Jin et al., 2008; Gelderblom et al., 2009). That is why, several 12/15-LOX inhibitors were synthesized for the targeting of the stroke (Sailer et al., 1998; Whitman et al., 2002; Deschamps et al., 2006; Kenyon et al., 2006; Rai et al., 2010). However, since most of these inhibitors are not selective, they could not reach the desired efficiency. Recently, van Leyen et al. developed a new compound named ML351 for its possible use in advanced biological models due to its superior properties such as solubility, microsomal stability, and crossing the BBB (Rai et al., 2014). Our study showed a decrease in infarct volume, neurological deficit score, and lipid peroxidation by administration of a more specific 12/15-LOX inhibitor, ML351, at the acute and subacute phases of ischemic stroke in mice which was compatible with the previous studies using different 12/15-LOX inhibitors in mice ischemic stroke (Jin et al., 2008; Yigitkanli et al., 2013; Liu X. et al., 2017; Karatas et al., 2018). This inhibitor may be regarded as an adjuvant therapeutic option with rtPA and other therapeutic strategies in cerebral ischemia (Karatas et al., 2018).

Here we show that 12/15-LOX inhibitor treatment effectively suppressed the activation of the inflammasome pathway, specifically the NLRP1 and NLRP3 complexes. I/R caused an increase in pro-inflammatory cytokines (IL-1beta, IL-6, TNF-alpha) at 6-h or 24-h of I/R, but not in the subacute (72-h) phases of stroke. 12/15-LOX inhibition significantly decreased IL-1beta, IL-6, and TNF-alpha protein levels at 24-h of stroke. While the early phase is characterized by an acute inflammatory response, the subacute phase is marked by a shift towards a more reparative and anti-inflammatory environment (Ran et al., 2021). Accordingly, anti-inflammatory cytokine (TGF-beta and IL-10) results displayed that TGF-beta was decreased after the stroke at all time points and only recovered at 72-h with 12/15-LOX inhibitor. IL-10 levels were increased prominently at 24-h of stroke with 12/15-LOX inhibition. These results suggest that the anti-inflammatory reaction of 12/15-LOX inhibition is mediated by IL-10 response at the acute phase while TGF-beta responds at the subacute phase of ischemia. This highlights the potential therapeutic benefits of ML351 in reducing neuroinflammation following I/R and the timing of targeted inflammatory reaction has utmost importance.

12/15-LOX enzyme causes peroxidation of membrane lipids under pathologic conditions such as ischemia. Oxidized lipids induce inflammation by activating NLRP3 (Liang et al., 2021). As a result, inhibition of the 12/15-LOX enzyme may play an important role in suppressing inflammation via the inflammasome pathway. The inflammation process that causes neuron and glial cell death in cerebral ischemia is mediated by protein complexes called inflammasomes. Inflammasomes are cytosolic multiprotein complexes and act as sensors to detect tissue damage. After a stimulus, the ASC protein, which is found in the nucleus under physiological conditions, is translocated into the cytoplasm. It oligomerizes in the cytoplasm, acting as a bridge between NLRP and procaspase-1, and assembles the complex (NLRP1 or NLRP3). It is known that activation of the inflammasomes causes the cleavage and release of pro-inflammatory cytokines (IL-1beta, IL-6) or alarmin (HMGB1) group proteins in the cell. Nowadays, it has been revealed that inflammasomes will be a new therapeutic target for ischemic stroke (Karatas et al., 2018; Alishahi et al., 2019). In the literature, new compounds were synthesized or employed to analyze the inhibitory effects of the inflammasome. Some of them are micro RNAs (miR223) (Bauernfeind et al., 2012), small molecules (MCC950) (Coll et al., 2015), nitric oxide (Mao et al., 2013), type I interferon (Guarda et al., 2011), nuclear factor erythroid-2 related factor 2 (Nrf2) (Liu X., et al., 2017), and polyphenolic compounds like curcumin (Ma et al., 2014; Ishrat et al., 2015; Chen P.Y. et al., 2023). However, the role of the inflammasomes in the inflammation for recovery of stroke has not been clarified. A study investigated the increased expression of NLRP1 immediately following stroke which was maintained at 12, 24, and 72-h (Fann et al., 2013). In our study, the NLRP1 immune signaling started to increase at 6-h of ischemia, and this increase was continued at 24-h and 72-h after I/R. The greatest induction of NLRP1 was observed at 24-h of the ischemia. This result is correlated with our ELISA results of pro-inflammatory cytokine levels, which were responsive mostly at 24-h following I/R. Interestingly, NLRP3 immunoreactivity dominantly increased in our stroke model compared to the NLRP1 inflammasome. The staining of both inflammasomes increased with I/R, which was significantly suppressed by 12/15-LOX inhibition. It is known that inflammasome activation includes procaspase-1 cleavage; accordingly, we showed that NLRP3-positive cells express caspase-1 supporting the formation of the inflammasome complex. In accordance with our results, it was reported that NLRP3 inflammasome-induced inflammation was reduced by lipoxygenase inhibition with cepharanthine at 24-h of cerebral I/R (Zhao et al., 2020). On the other hand, recent study findings were incompatible with the previous literature. They report that ischemic brain injury was not reduced by specific inhibition of NLRP3 with MCC950 or NLRP3^−/−^ in mice (Lemarchand et al., 2019). These conflicting results clearly show that stroke pathophysiology has a complex nature, and targeting only one pathway may not be sufficient to decrease the ischemic damage. In addition to its anti-inflammatory properties, 12/15-LOX has been implicated in other pathophysiological processes in stroke, such as oxidative stress and neuronal apoptosis (Liu Y. et al., 2017; Karatas and Cakir-Aktas, 2019; Kursun et al., 2022). That is why our study highlights the importance of considering multiple pathways and their interactions in the development of stroke treatment. This research also provides valuable insights into the complex nature of inflammatory responses and offers potential avenues for future research and treatment options.

Furthermore, in our study, the cellular source of the inflammasome complex showed that NLRP1 and NLRP3 were expressed in neurons. This finding corroborates the recent literature suggesting the importance of neuronal inflammasome activation in stroke pathophysiology, including Gong et al.’s study, which indicated that the NLRP3 inflammasome expressed by microglia initially was followed by microvascular endothelial cells and neurons, but principally in neurons 24-h after cerebral I/R (Gong et al., 2018). Others have also shown that NLRP3 expression increases specifically in neurons after 4-h, 8-h, and 24-h following transient MCAo (Franke et al., 2021). In our study, we found that NLRP3 expression was shifted from neurons to non-neuronal cells at 72-h of I/R. Therefore, the cellular source of NLRP3 may vary significantly according to distinct pathologies, such as migraine, permanent or transient cerebral ischemia, as well as the process of stroke-induced brain damage. Interestingly, we showed that increased NLRP3 signaling was present in 12/15-LOX-positive cells, which were suppressed by ML351, which indicated a direct interaction of the inflammasome and lipoxygenase pathways. It is known that 12/15-LOX activity leads to neuronal death. Recently, an increasing number of studies have reported that the activation of inflammation-related signaling pathways is closely connected with ferroptosis (Chen Y. et al., 2023). Ferroptosis is an iron-dependent regulated cell death driven by excessive lipid peroxidation. Studies have indicated that increased lipid peroxidation can activate multiple inflammatory pathways including inflammasomes, while proinflammatory cytokines, in turn, aggravate intracellular oxidative stress and excessive lipid peroxidation. 12/15-LOX initiates the peroxidation of phospholipids not only in the plasma membrane but also within the mitochondrial membrane, leading to the formation of membrane defects. This facilitates the translocation of mitochondrial DNA (mtDNA) from the matrix to the cytoplasm. This extramitochondrial milieu exposes mtDNA to excessive oxidative stress. The outcome of the oxidative modification of mtDNA enhances its interaction with NLRP3, thereby stimulating the assembly of the NLRP3 inflammasome protein complex which eventually may lead to neuronal cell death (van Leyen et al., 2006; Qiu et al., 2022). Further studies focusing on the crosstalk between lipid peroxidation, inflammasome activation, ferroptosis, and other cell death pathways may shed light on the pathophysiological mechanism of each process and might provide novel therapeutic targets for relevant diseases. Overall, our findings suggest that targeting NLRP1 or NLRP3 inflammasome activation by 12/15-LOX inhibition may be a promising approach for reducing inflammation and improving outcomes in I/R injury.

Additionally, this study emphasizes the significance of understanding the cellular sources and expression patterns of key inflammatory molecules at different stages of stroke progression. By identifying these factors, we may have a better understanding of the underlying mechanisms driving stroke pathophysiology. Opposite studies may be related to the complex and dynamic nature of the inflammatory response in stroke, which involves multiple cell types and signaling pathways. Further research is needed to elucidate the precise mechanism underlying the effects of 12/15-LOX inhibition on post-stroke inflammation and to explore its potential as a therapeutic target for stroke. Therefore, a comprehensive understanding of its role in stroke pathogenesis is crucial for developing effective interventions.

Limitations of our study include the low number of mice used in some experiments. To overcome this limitation, we tried to increase the accuracy of the results by obtaining at least 3 images from consecutive periinfarct areas and repeating ELISA studies 2–3 times. Yet we obtained statistically significant results. Another one is studying only brain tissue for ELISA analysis; serum samples would be valuable to compare brain tissue results with circulating cytokines. However, serum levels of inflammatory cytokines may not represent brain inflammation accurately as there may be other systemic confounding factors.

Our results provide new insights into the role of 12/15-LOX in inflammasome signaling and neuronal damage. The activation of the NLRP3 inflammasome in 12/15-LOX-positive cells may contribute to the inflammatory response and neural damage observed in stroke. Targeting this pathway may represent a novel therapeutic strategy for stroke prevention and treatment. In addition, our findings highlight the need for further investigation into the mechanisms underlying inflammasome activation in neurons and their contribution to neurological disorders. Overall, our study sheds light on the complex interplay between inflammation, oxidative stress, and neuronal damage in stroke pathophysiology, and opens new avenues for future research. Therefore, further research is needed to fully understand the role of 12/15-LOX in stroke-induced neuroinflammation. In addition, the development of specific inhibitors targeting 12/15-LOX may provide a potential therapeutic strategy for stroke patients. It is also important to note that neuroinflammation, oxidative stress, and lipid peroxidation are not only involved in stroke but also in other neurological disorders such as Alzheimer’s disease and Parkinson’s disease. Thus, understanding the mechanisms underlying lipid peroxidation and its associated pathways may have broader implications for the treatment of various neurological diseases. Overall, continued research on lipid peroxidation and its effects on neuroinflammation will contribute to a better understanding of the pathophysiology of stroke and other neurological disorders, ultimately leading to improved treatments and outcomes for patients.

## 5. Conclusion

In summary, these results indicate that one of the possible mechanisms of 12/15-LOX inhibition involves the suppression of neuroinflammation in both the acute and subacute phases of cerebral ischemia/recanalization by suppressing inflammasomes and inflammasome-related proteins. These findings also suggest that 12/15-LOX inhibitors may be a treatment option for stroke therapy or other diseases characterized by neuroinflammation.

## Data availability statement

The original contributions presented in the study are included in the article/supplementary material, further inquiries can be directed to the corresponding author.

## Ethics statement

The animal study was approved by Hacettepe University, Animal Experiments Ethics Committee (Approval No: 2016/27–04). The study was conducted in accordance with the local legislation and institutional requirements.

## Author contributions

CCA: Conceptualization, Data curation, Investigation, Methodology, Software, Writing – original draft, Writing – review & editing. EB: Conceptualization, Investigation, Writing – review & editing, Supervision. MY: Investigation, Writing – review & editing, Conceptualization, Supervision. KL: Conceptualization, Investigation, Writing – review & editing. HK: Conceptualization, Funding acquisition, Project administration, Supervision, Writing – original draft, Writing – review & editing, Investigation, Methodology.
